# Transactions of the Medical Association of Southern Central New York, at the Ninth Annual Meeting, Held at Elmira, June 5th, 1855

**Published:** 1855-11

**Authors:** 


ART. XIII.—*Transactions of the Medical Association of Southern Central New York, at the Ninth Annual Meeting, held at Elmira, June 5th,* 1855.

The annual appearance of a handsome volume of transactions from an association entirely voluntary, and dependent on the zeal of its members only for its success, owing nothing to state aid, and having no possible object except the mutual improvement of its members, and the elevation of the dignity of the profession, is, in itself, a matter well worth our notice and attention. When we add to this that this local body produces a series of papers of intrinsic merit usually far exceeding those of the parent state society, and always richly repaying perusal, we say a good deal—and that with justice—for the character of the Southern Central Association.

The papers in this volume are numerous and from equally numerous sources, a feature indicative of vitality. Among the names with which we are familiar, we notice those of Prof. Caleb Green, of Homer, Prof. Hyde, of Cortlandville, and Dr. John G. Orton, of Binghampton, as attached to papers of much interest. The growing demand upon our space is our apology for this entirely insufficient notice of this energetic and progressive association.

